# Hospital volume-mortality association after esophagectomy for cancer: a systematic review and meta-analysis

**DOI:** 10.1097/JS9.0000000000001185

**Published:** 2024-02-14

**Authors:** Jie Di, Xiao-Shi Lu, Min Sun, Zhe-Ming Zhao, Chun-Dong Zhang

**Affiliations:** a Clinical Medicine; b Central Laboratory; c Department of Surgical Oncology, The Fourth Affiliated Hospital of China Medical University, Shenyang; d Department of General Surgery, Taihe Hospital, Hubei University of Medicine, Shiyan, People’s Republic of China

**Keywords:** esophageal cancer, hospital surgical case volume, postoperative mortality, volume–outcome

## Abstract

**Background:**

Postoperative mortality plays an important role in evaluating the surgical safety of esophagectomy. Although postoperative mortality after esophagectomy is partly influenced by the yearly hospital surgical case volume (hospital volume), this association remains unclear.

**Methods:**

Studies assessing the association between hospital volume and postoperative mortality in patients who underwent esophagectomy for esophageal cancer were searched for eligibility. Odds ratios were pooled for the highest versus lowest categories of hospital volume using a random effects model. The dose-response association between hospital volume and the risk of postoperative mortality was analyzed. The study protocol was registered with PROSPERO.

**Results:**

Fifty-six studies including 385 469 participants were included. A higher-volume hospital significantly reduced the risk of postesophagectomy mortality by 53% compared with their lower-volume counterparts (odds ratio, 0.47; 95% CI: 0.42–0.53). Similar results were found in subgroup analyses. Volume–outcome analysis suggested that postesophagectomy mortality rates remained roughly stable after the hospital volume reached a plateau of 45 esophagectomies per year.

**Conclusions:**

Higher-volume hospitals had significantly lower postesophagectomy mortality rates in patients with esophageal cancer, with a threshold of 45 esophagectomies per year for a high-volume hospital. This remarkable negative correlation showed the benefit of a better safety in centralization of esophagectomy to a high-volume hospital.

## Introduction

Highlights

- The likelihood of postesophagectomy mortality has been reduced by 53% in high-volume hospitals.
- An annual esophagectomy volume of at least 45 may be defined as a high hospital volume.

Esophageal cancer accounts for 3% of all cancer cases and is the seventh most-diagnosed cancer and sixth most common cause of cancer-related death worldwide, with East Asia contributing 59.2% of all cases and China alone accounting for 53.7%^1^. No randomized clinical trials have yet confirmed any advantages of minimally invasive esophagectomy in terms of outcomes such as complications and mortality, and open surgery thus remains the dominant and preferred option^2–4^. Postoperative mortality is a crucial metric for assessing the safety of surgery^5,6^, with rates of 0.6–23.8% in patients following radical esophagectomy for esophageal cancer, depending on the region and study period^7,8^. There have been significant advancements in the early detection and accurate diagnosis of esophageal cancer and precursor lesions (e.g. Barrett’s esophagus), including endoscopic techniques and the introduction of techniques such as machine learning in diagnostic procedures^1,9^, while advances in rehabilitation, intensive care, and perioperative care techniques have also reduced postoperative mortality^10–12^. Numerous studies have also suggested an association between hospital volume and postoperative mortality^13^; however, uncertainty persists regarding the relationship between the number of surgical procedures performed in hospitals and the risk of postoperative mortality in patients with esophageal cancer undergoing esophagectomy.

Nevertheless, this volume–outcome association may be significant in terms of the high-risk and low-volume of esophagectomy procedures for cancer. It has therefore been suggested that concentrating these treatments with high-volume providers could enhance patient outcomes in general. This study thus carried out a systematic review and meta-analysis of the relevant literature to investigate the volume–outcome effect of hospital surgical case volume on the risk of postesophagectomy mortality in these patients, with the aim of establishing a threshold for the minimum annual surgical volume for high-volume hospitals.

## Methods

This systematic review was conducted following the Meta-analysis of Observational Studies in Epidemiology (MOOSE) and Preferred Reporting Items for Systematic Reviews and Meta-analysis (PRISMA) guidelines (Supplemental Digital Content 1, http://links.lww.com/JS9/B903, Supplemental Digital Content 2, http://links.lww.com/JS9/B904)^14–16^. Each quality assessment was based on AMSTAR 2 (Supplemental Digital Content 3, http://links.lww.com/JS9/B905)^17^, which is highly descriptive and consistent. The protocol was registered with the International Prospective Register of Systematic Reviews (PROSPERO) prior to conducting this systematic review.

### Eligibility criteria

The eligibility criteria were relevant cohort studies investigating the relationship between hospital surgical case volume for esophagectomy and postoperative mortality in patients undergoing surgery for esophageal cancer. Postoperative mortality was defined in this research as death occurring while hospitalized, irrespective of the length of hospital stay, and death occurring after hospital discharge within 30 days after surgery. The exclusion criteria were case reports, reviews, and studies that lacked enough data; studies with no short-term mortality data; studies with 60-day or 90-day mortality data only; studies with no clear definition of operative death; studies where it was not possible to distinguish between data for benign illness and esophageal cancer; studies with a mixture of esophageal and other cancer types; studies including nonsurgical patients; studies including endoscopic treatments; studies with surgeon-volume data only or hospital-type data only; studies that lacked or had ambiguous reference groups; and studies with continuous data only.

### Data sources and search strategy

Two authors independently searched PubMed and Embase until 9 November 2023. The search terms included esophageal cancer, esophagectomy, hospital surgical volume, and their variants. The search strategies were shown (Supplemental Data Table 1, Supplemental Digital Content 4, http://links.lww.com/JS9/B906). We further searched the reference lists of relevant articles and conference abstracts for potential unpublished studies.

### Study selection

After conducting a systematic search to identify duplicate studies, the two authors evaluated the qualities of the remaining studies and any disagreements were resolved by a third reviewer. Relevant studies were initially screened based on their titles and abstracts, and the full-text was then read to confirm its relevance. Non-English studies were translated and read by translation software, to avoid missing potentially relevant studies.

### Data extraction

The two authors each extracted data from the articles following a well-formulated procedure. Data on each study’s first author, publication year, study design, location, study period, number of patients who underwent esophagectomy for esophageal cancer, number of hospitals, hospital volume category (annual surgical cases per year), postoperative mortality, definition of postoperative mortality, and covariates were all obtained and examined by two other reviewers. Any disagreements were settled by discussion and agreement.

### Quality assessment

The two authors independently evaluated the qualities of the included studies using the Newcastle–Ottawa Scale (NOS) for cohort design. All studies were evaluated regarding participant selection and measurement of exposure, comparability, assessment of outcomes, and adequacy of follow-up. The studies were then rated as high (7–9), moderate (4–6), or low quality (0–3).

### Statistical analysis

We collected the odds ratio (OR) and 95% CIs to analyze the relationship between the number of hospital esophagectomy procedures (highest vs. lowest) and the risk of postoperative mortality in patients with esophageal cancer who underwent esophagectomy. The reference group was the hospital with the lowest volume. A random effects model was adopted because of unavoidable clinical heterogeneity. Heterogeneity was assessed using the *Q* statistic, with *I* ^2^ values <25%, 25–50%, and >50% indicating low, moderate, and high heterogeneity, respectively. Publication bias was assessed using funnel plots with Begg’s and Egger’s tests. Trim and fill method was further applied to estimate potential missing studies, for adjustment of the funnel plot and the pooled ORs were recalculated^18^. *P*<0.05 was considered statistically significant. All statistical analyses were performed using R 4.2.2 and Stata 13.0 softwares.

#### Subgroup analyses

To further confirm the robustness of the findings, we performed multiple subgroup analyses in terms of study period (1984–2004 and 2005–2019), country (Eastern and Western), sample size (≤5000 and ≥5000), hospital number (≤100, ≥100, and unknown), volume grouping (dichotomies, tertiles, quartiles, quintiles, sextiles, and seventh percentiles), adjusted ORs (yes and no), NOS quality assessments (total score=9 and <9), and NOS quality assessments (comparability score=2 and <2).

#### Sensitivity analyses

Sensitivity analyses were conducted using the leave-one-out method (omitting one trial each time and repeating the meta-analysis) to further test the robustness of the findings.

#### Volume–outcome analysis

Additionally, we examined the volume–outcome association between the number of esophagectomies performed at the hospital and the likelihood of postoperative mortality in patients undergoing esophagectomy for esophageal cancer. As reported previously, a minimum of three quantitative categories of hospital volume, the number of postoperative deaths, the overall number of participants, and ORs with 95% CIs were needed for this procedure^19^. If the median (or mean) value was not indicated, we estimated it based on the midpoint between the upper and lower limits: the lower boundary was assumed to be zero if it was open-ended; otherwise, the median value was assumed to be 1.5 times the lower boundary if the upper boundary was open-ended, as described previously^20^. The volume–outcome relationship was compared with a linear trend relationship, with *P≥*0.05 indicating a linear relationship and *P*<0.05 indicating a nonlinear trend. To demonstrate the connection between the two, we created a scatter plot showing the distribution between the number of hospitalized surgical procedures (*X*-axis) and the postoperative mortality rate (*Y*-axis) in patients with esophageal cancer undergoing esophagectomy.

## Results

**Figure 1:**
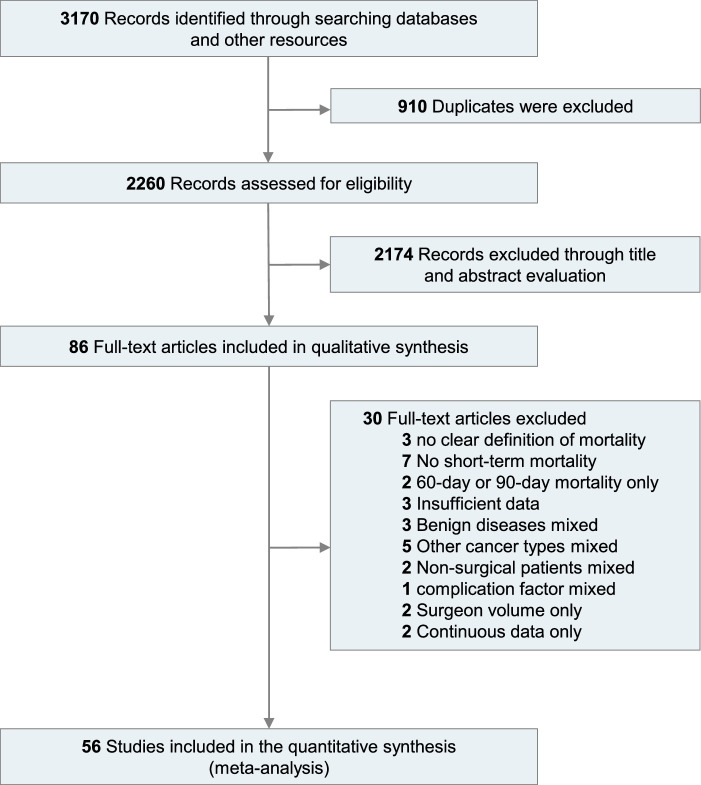
Flowchart of literature search and study selection.

The initial search identified 3170 potential studies. After removing 910 duplicates, 2260 studies remained. Among these, 2174 were discarded after reviewing the titles and abstracts and 30 were removed after reviewing the full-text articles. Finally, 56 cohort studies were included after reviewing the full texts^7,8,21–74^. Details of the literature search are shown in Figure 1.

### Characteristics of included studies

The characteristics of the 56 included studies are summarized in Supplemental Data Table 2 (Supplemental Digital Content 4, http://links.lww.com/JS9/B906). The numbers of patients in these studies ranged from 340–31 380, with a total of 385 469 patients. All studies were retrospective cohort studies. Five studies were from Eastern countries and the remaining 51 were from Western countries. The postoperative mortality rates ranged from 0.6–23.8%. The hospital volume cutoffs, ORs, and adjusted factors are presented in Supplemental Data Table 3 (Supplemental Digital Content 4, http://links.lww.com/JS9/B906). The NOS for quality assessment of the included studies is presented in Supplemental Data Table 4 (Supplemental Digital Content 4, http://links.lww.com/JS9/B906).

### Hospital volume and risk of postoperative mortality

**Figure 2:**
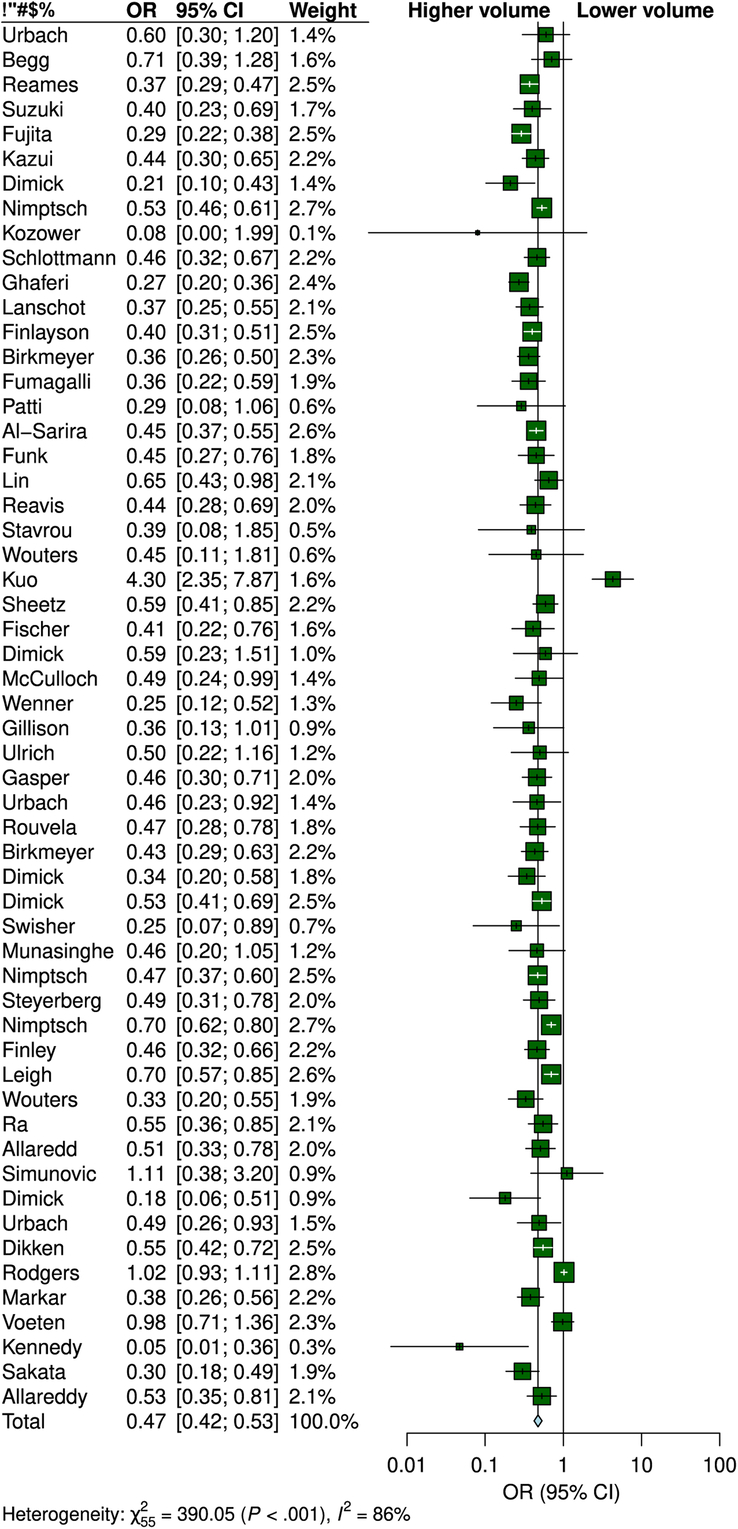
Forest plot of association between hospital surgical case volume per year and the risk of postoperative mortality among esophageal cancer patients undergoing esophagectomy according to volume grouping.

Fifty-six studies including 385 469 participants with esophageal cancer were included in the quantitative analysis. The risk of postoperative mortality among esophageal cancer patients undergoing esophagectomy was reduced by 53% in higher-volume hospitals compared with their lower-volume counterparts (OR, 0.47; 95% CI: 0.42–0.53) (Fig. 2).

### Subgroup analyses

Multiple subgroup analyses were conducted to further confirm the robustness of the findings. The risk of postesophagectomy mortality was significantly reduced in higher-volume hospitals compared with lower-volume hospitals by 52% in Western countries (OR, 0.48; 95% CI: 0.43–0.55), 61% in Eastern countries (OR, 0.39; 95% CI: 0.29–0.54), 51% during the period of 1984–2004 (OR, 0.49; 95% CI: 0.40–0.61), 54% during the period of 2005–2019 (OR, 0.46; 95% CI: 0.40–0.52), 51% in studies including ≤100 hospitals (OR, 0.49; 95% CI: 0.33–0.72), 55% in studies including ≥100 hospitals (OR, 0.45; 95% CI: 0.40–0.50), 51% in studies with a sample size ≤5000 (OR, 0.49; 95% CI: 0.40–0.60), 53% in studies with a sample size ≥5000 (OR, 0.47; 95% CI: 0.42–0.53), 51% in adjusted ORs (OR, 0.49; 95% CI: 0.42–0.57), 51% in studies with NOS total score=9 (OR, 0.49; 95% CI: 0.44–0.56), 65% in studies with NOS total score <9 (OR, 0.35; 95% CI: 0.25–0.49), 51% in studies with NOS comparability score=2 (OR, 0.49; 95% CI: 0.44–0.56), and 65% in studies with NOS comparability score <2 (OR, 0.35; 95% CI: 0.24–0.50) (Supplemental Data Figures 1–8, Supplemental Digital Content 4, http://links.lww.com/JS9/B906; Table 1).

**Table 1:** Subgroup analyses of volume effect on postoperative mortality in esophageal cancer patients undergoing esophagectomy.

					Test of heterogeneity	
Subgroup	No. of patients	No. of studies	Odds ratio (95% CI)	*P*	*I* ^2^ value	*P*
Total	385 469	56	0.47 (0.42–0.53)	<0.01	86%	<0.01
Study period						
1984–2004	77 867	28	0.49 (0.40–0.61)	<0.01	89%	<0.001
2005–2019	299 130	26	0.46 (0.40–0.52)	<0.01	76%	<0.001
Country						
Eastern	94 801	5	0.39 (0.29–0.54)	<0.01	67%	0.02
Western	290 668	51	0.48 (0.43–0.55)	<0.01	86%	<0.001
Sample size						
<5000	62 929	33	0.49 (0.40–0.60)	<0.01	84%	<0.001
≥5000	322 540	22	0.47 (0.42–0.53)	<0.01	75%	<0.001
Unknown	Not applicable	1	0.27 (0.20–0.36)	<0.01	Not applicable	Not applicable
Hospital number						
<100	25 331	16	0.49 (0.33–0.72)	<0.01	82%	<0.001
>100	265 801	26	0.45 (0.40–0.50)	<0.01	66%	<0.001
Unknown	94 337	14	0.51 (0.41–0.63)	<0.01	91%	<0.001
Volume grouping						
Dichotomies	74 118	15	0.55 (0.39–0.76)	<0.01	80%	<0.001
Tertiles	48 026	16	0.47 (0.38–0.57)	<0.01	90%	<0.001
Quartiles	39 034	9	0.49 (0.34–0.69)	<0.01	69%	0.001
Quintiles	147 667	13	0.45 (0.39–0.52)	<0.01	60%	0.003
Sextiles	31 380	1	0.29 (0.22–0.38)	<0.01	Not applicable	
Seventh percentiles	45 244	2	0.37 (0.26–0.54)	<0.01	32%	0.23
Adjusted OR						
Yes	245 780	41	0.49 (0.42–0.57)	<0.01	88%	<0.001
Unknown	139 689	15	0.43 (0.37–0.50)	<0.01	23%	0.2
NOS quality assessments						
Total score=9	379 811	10	0.49 (0.44–0.56)	<0.01	87%	<0.001
Total score <9	5658	46	0.35 (0.25–0.49)	<0.01	40%	0.09
NOS quality assessments						
Comparability score=2	380 132	9	0.49 (0.44–0.56)	<0.01	87%	<0.001
Comparability score <2	5337	47	0.35 (0.24–0.50)	<0.01	46%	0.06

CI, confidence interval; No., number; OR, odds ratio.

In terms of volume groupings, higher-volume hospitals significantly reduced the risk of postesophagectomy mortality compared with their lower-volume counterparts by 45% in dichotomous (OR, 0.55; 95% CI: 0.39–0.76), 53% in tertiles (OR, 0.47; 95% CI: 0.38–0.57), 55% in quintiles (OR, 0.45; 95% CI: 0.39–0.52), 51% in quartiles (OR, 0.49; 95% CI: 0.34–0.69), 71% in sextiles (OR, 0.29; 95% CI: 0.22–0.38), and 63% in seventh percentiles (OR, 0.37; 95% CI: 0.26–0.54) (Supplemental Data Figure 6, Supplemental Digital Content 4, http://links.lww.com/JS9/B906; 1). Overall, subgroup analyses revealed that these results were robust.

### Sensitivity analyses

Sensitivity analyses using the leave-one-out method achieved similar overall combined ORs, ranging from 0.46 (95% CI: 0.42–0.51) to 0.48 (95% CI: 0.43–0.54) (Supplemental Data Figure 9, Supplemental Digital Content 4, http://links.lww.com/JS9/B906), indicating that the current findings were robust.

### Volume–outcome analysis

**Figure 3:**
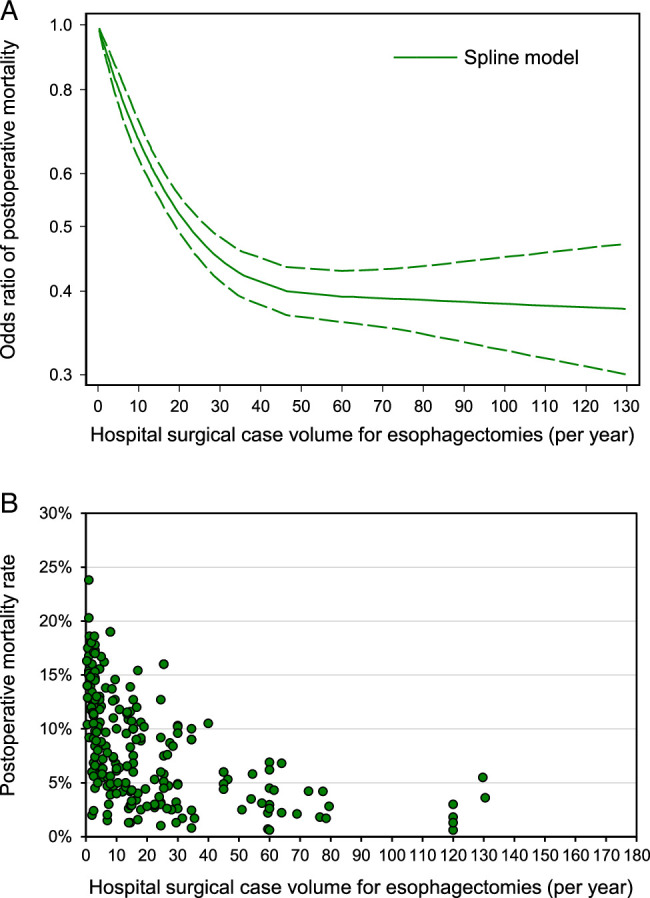
A. Volume–outcome analysis of association between hospital surgical case volume per year and the risk of postoperative mortality among esophageal cancer patients undergoing esophagectomy. Middle green line: fit curve of odds ratio; upper green line: upper 95% CI; lower green line: lower 95% CI. B. Scatter plot of distribution between median or mean hospital surgical case volume per year in each category from 56 studies and corresponding mortality rates among esophageal cancer patients undergoing esophagectomy.

Forty studies were included in the volume–outcome analysis between hospital surgical case volume and the risk of postesophagectomy mortality in esophageal cancer patients. The volume–outcome graph suggested that this correlation was not linear (Fig. 3), and the risk of postesophagectomy mortality remained consistent or declined slightly after the hospital volume reached 45 esophagectomies per year.

Furthermore, 44 studies were included to assess the relationship between hospital surgical case volume and postoperative mortality rates (Fig. 3). Similarly, the postoperative mortality rates showed a rapid decline from 23.8% to 6.0% until the hospital volume reached a threshold of around 45 esophagectomies per year. Moreover, the postoperative mortality rate stabilized at around 5% after reaching 70 esophagectomies per year.

### Publication bias

**Figure 4:**
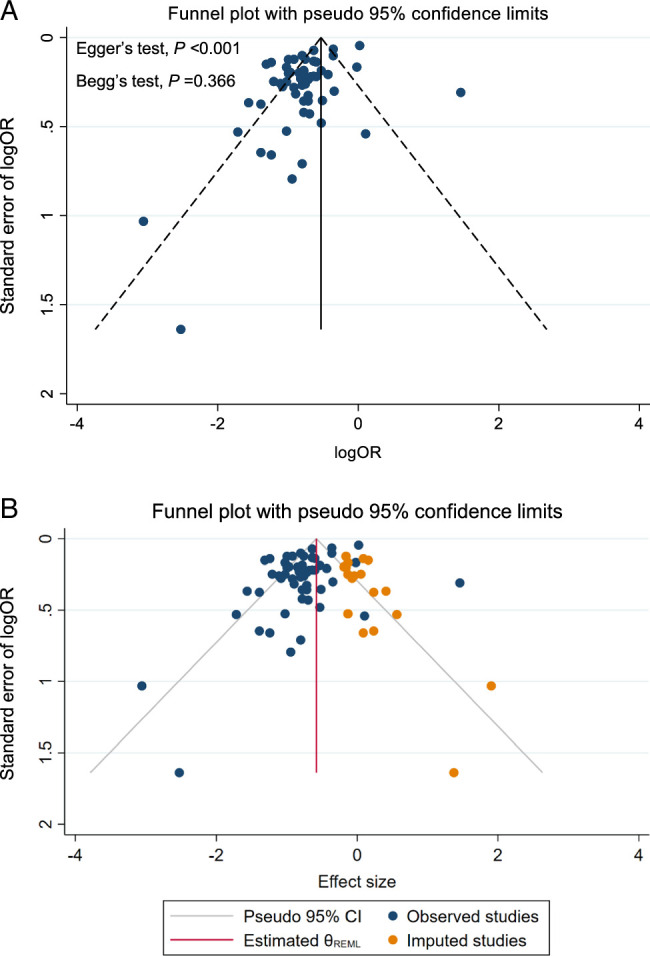
A. Funnel plot for publication bias of association between hospital surgical case volume per year and the risk of postoperative mortality among esophageal cancer patients undergoing esophagectomy. B. Funnel plot for publication bias after processing trim-and-fill analysis.

Fifty-six studies were included in the quantitative meta-analysis. There was no clear evidence of publication bias based on Begg’s (*P*=0.339) test; however, Egger’s test (*P*<0.001) suggested the existence of publication bias (Fig. 4A). In an attempt to minimize the effects of publication bias, we processed the funnel plots using the trim-and-filling method, which yielded results after supplementing the literature. The trim-and-fill analysis revealed 18 potential studies were absent. After filling those 18 trials, the refitted data (effect size, 0.56; 95% CI: 0.50–0.63) remained consistent with the above findings (Fig. 4B).

## Discussion

Esophagectomy remains a high-risk procedure in patients with esophageal cancer, with a high postoperative mortality rate. Here, we extracted 232 groups of mortality data, of which 163 groups (70.3%) showed postoperative mortality rates >5%, with a highest mortality rate of 23.8%^8^. We therefore aimed to investigate the relationship between hospital surgical volume and postoperative mortality in patients with esophageal cancer undergoing esophagectomy. Importantly, there was a 53% reduction in the risk of postoperative mortality in higher-volume hospitals compared with their lower-volume counterparts. Furthermore, we identified a high-volume-hospital threshold of around 45 esophagectomies per year.

A previous meta-analysis reported a substantial association between hospital volume and postesophagectomy mortality in 2004, with a cutoff value of 20 esophagectomies per year; however, the reason for applying this threshold was not explained, and the analysis only included 13 studies during the period from 1990 to 2003^75^. Another meta-analysis found a similar association between hospital volume and postesophagectomy mortality without identifying a threshold for high-volume hospitals^76^. The present study included a total of 56 studies and showed that the risk of postesophagectomy mortality was significantly reduced by 53% in higher-volume hospitals. Moreover, for the first time, we identified a threshold of 45 esophagectomies per year for high-volume hospitals.

Although many studies have supported an association between hospital volume and postesophagectomy mortality in patients with esophageal cancer, the causal roles of hospital volume remain unclear, including the effect of surgeon volume on patient survival^77,78^. A previous multicenter study identified the capacity to revive patients from surgical problems as an essential factor that could increase the likelihood of postoperative survival in cancer patients^12^. High-volume hospitals may also be able to reduce postoperative mortality by using more advanced intensive care units/techniques and medical facilities^11,79^. There are additional conjectures that could potentially highlight the association between hospital volume and postoperative mortality. Accordingly, implementing the ‘focused-factory’ model, which entails standardizing the processing of therapeutic programs, can successfully enhance both costs and outcomes, and hospitals with high patient volumes are more likely to use this work paradigm^71,80^. Notably, in addition to operating considerations, medical treatments during and after complex procedures also have crucial influences on postoperative mortality^69^. Furthermore, patients in high-volume hospitals are more likely to undergoing more perioperative monitoring and have higher quality nursing, which may also reduce the risk of postoperative mortality^34,81^. Accordingly, a higher success rate and a lower failure-to-rescue rate have also been attributed to the educational qualifications of nurses^82^, while a recent study indicated that high-volume anesthesiology care was also linked to lower morbidity^83^.

The strengths of this study included prospectively registered on PROSPERO prior to conducting this systematic review, conducted in according to the Cochrane and PRISMA guidelines (Supplemental Digital Content 1, http://links.lww.com/JS9/B903). Moreover, this study included 56 cohort studies with a total of 385 469 patients. For the first time, this study identified the threshold of 45 esophagectomies per year for a high-volume hospital. However, this study still had several limitations. Firstly, the current study primarily focused on the association between hospital volume and postesophagectomy mortality, overlooking the potential role of single surgeon volume as a significant confounder associated with postoperative mortality^84–87^. Secondly, since all data originated from retrospective cohorts, perspective studies are needed to confirm current findings. Thirdly, data showed substantial heterogeneity and a publication bias was evident through the Egger’s test; anyway, the main findings remained robust by the trim-and-filling method and in subgroup analyses. Lastly, the current evidence should be approached cautiously due to reliance on data from administrative databases and the presence of clustering bias due from multicenter data retrieval.

## Conclusions

Higher-volume hospitals had significantly lower postesophagectomy mortality rates in patients with esophageal cancer, with a threshold of 45 esophagectomies per year for a high-volume hospital. This remarkable negative correlation showed the benefit of a better safety in centralization of esophagectomy to a high-volume hospital.

## Ethical approval

Not applicable.

## Sources of funding

This study was supported by the Scientific Study Project for Institutes of Higher Learning, Ministry of Education, Liaoning Province (JYTMS20230108), and the Young Backbone Talents of China Medical University (RXXM202302). Min Sun was supported by the Natural Science Foundation of Hubei Province of China (2023AFB1008).

## Author contribution

J.D., X.-S.L., Z.-M.Z., and C.-D.Z.: conceived and designed the experiments; J.D. and X.-S.L.: analyzed the data; J.D., X.-S.L., and M.S.: contributed reagents/materials/analysis; J.D., X.-S.L., Z.-M.Z., M.S., and C.-D.Z.: wrote the manuscript. All authors have read and approved the final manuscript.

## Conflicts of interest disclosure

The authors declare no conflicts of interest.

## Research registration unique identifying number (UIN)

1. Name of the registry: PROSPERO database.
2. Unique identifying number or registration ID: CRD42023465897.
3. Hyperlink to your specific registration (must be publicly accessible and will be checked): https://www.crd.york.ac.uk/prospero/display_record.php?ID=CRD42023465897.

## Guarantor

Chun-Dong Zhang.

## Data availability statement

All data generated and analyzed during this study are included in this article. The data supporting the findings of this study are available from the corresponding author upon reasonable request.

## Supplementary Material
