# Supplementary figures and images for: Hospital volume-mortality association after esophagectomy for cancer: a systematic review and meta-analysis
Source: Int J Surg. 2024 Feb 14;110(5):3021–9. doi: 10.1097/JS9.0000000000001185 (PMC11093504; doi:10.1097/JS9.0000000000001185)

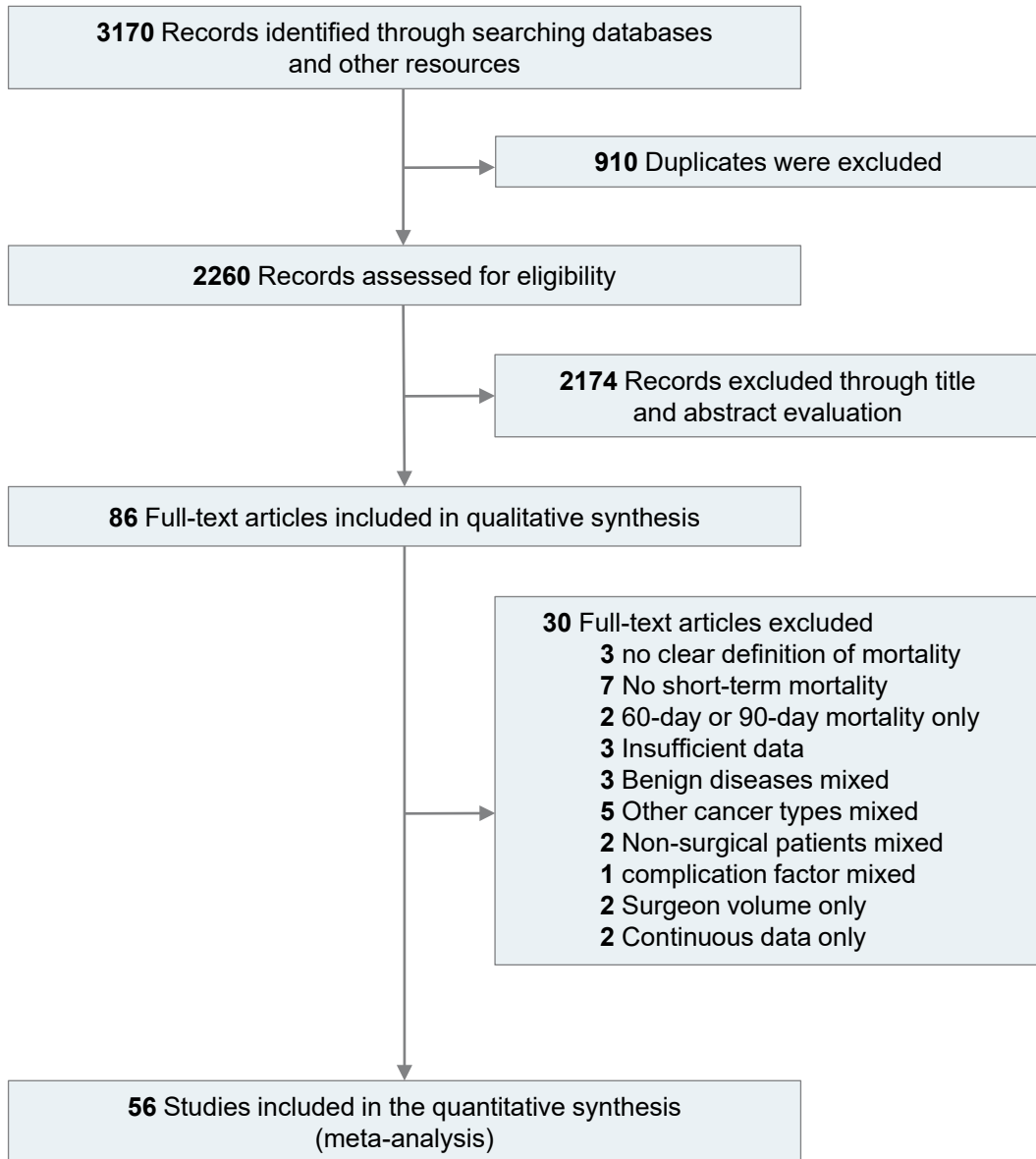

Supplement: Supplementary file 2 [file js9-110-3021-s002.pdf]
