# Supplementary material for: Hospital volume-mortality association after esophagectomy for cancer: a systematic review and meta-analysis
Source: Int J Surg. 2024 Feb 14;110(5):3021–9. doi: 10.1097/JS9.0000000000001185 (PMC11093504; doi:10.1097/JS9.0000000000001185)
Supplement: Supplementary file 4 [file js9-110-3021-s004.docx]

**Supplemental Table 1.** Search strategy.

| **PubMed** | |
| --- | --- |
| #1 | "esophageal neoplasms"[Title/Abstract] OR "cancer of esophagus"[Title/Abstract] OR "cancer of the esophagus"[Title/Abstract] OR "esophageal cancer"[Title/Abstract] OR "esophagus cancer"[Title/Abstract] OR "esophagus neoplasm"[Title/Abstract] OR "neoplasms esophageal"[Title/Abstract] OR "esophagectomy"[Title/Abstract] |
| #2 | ((((((((("volume"[Title/Abstract] NOT "tidal"[Title/Abstract]) NOT "hemodialysis"[Title/Abstract]) NOT "stroke"[Title/Abstract]) NOT "lung"[Title/Abstract]) NOT "pressure"[Title/Abstract]) NOT "platelet"[Title/Abstract]) NOT "blood"[Title/Abstract]) NOT "brain"[Title/Abstract]) NOT "hematoma"[Title/Abstract]) NOT "hemofiltration"[Title/Abstract] |
| #3 | #1 AND #2 |
| **Embase** | |
| #1 | ' esophageal neoplasms ':ab,ti OR ' esophageal cancer ':ab,ti OR ' esophagus cancer ':ab,ti OR ' esophagus neoplasm ':ab,ti OR 'esophageal tumor':ab,ti OR ' neoplasms esophageal ':ab,ti OR ' esophagectomy ':ab,ti |
| #2 | volume:ab,ti NOT tidal:ab,ti NOT hemodialysis:ab,ti NOT stroke:ab,ti NOT lung:ab,ti NOT pressure:ab,ti NOT platelet:ab,ti NOT blood:ab,ti NOT brain:ab,ti NOT hematoma:ab,ti NOT hemofiltration:ab,ti |
| #3 | #1 AND #2 AND [embase]/lim |

**Supplementary Table 2** Clinical characteristics of patients undergoing esophagectomy from 56 included studies.

| No | Study | Country | Period | Study design | Number of | Number of | Operation types | Hospital volume | Odds ratio | POM | Definition of |
| --- | --- | --- | --- | --- | --- | --- | --- | --- | --- | --- | --- |
|  |  |  |  |  | patients | hospitals |  | (cases/year) | (95% CI) | (death/total) | POM |
| 1 | Reames BN 2014 | United States | 2000-2001 | Retrospective | 6315 | 1559 | esophagectomy | ＜2 | 2.25 (1.57 - 3.23) | 16.76% | 30-day |
|  |  |  |  |  |  |  |  | ＞12 | 1 (reference) | 8.9% |  |
|  |  | United States | 2002-2003 | Retrospective | 6046 | 1477 | esophagectomy | ＜2 | 1.92 (1.36 - 2.70) | 15.37% | 30-day |
|  |  |  |  |  |  |  |  | ＞12 | 1 (reference) | 9.16% |  |
|  |  | United States | 2004-2005 | Retrospective | 5464 | 1310 | esophagectomy | ＜2 | 3.18 (2.41 - 4.18) | 15.01% | 30-day |
|  |  |  |  |  |  |  |  | ＞17 | 1 (reference) | 5.8% |  |
|  |  | United States | 2006-2007 | Retrospective | 5204 | 1216 | esophagectomy | ＜2 | 2.41 (1.66 - 3.52) | 15.14% | 30-day |
|  |  |  |  |  |  |  |  | ＞17 | 1 (reference) | 7.49% |  |
|  |  | United States | 2008-2009 | Retrospective | 6601 | 1229 | esophagectomy | ＜3 | 3.68 (2.66 - 5.11) | 13.94% | 30-day |
|  |  |  |  |  |  |  |  | ＞18 | 1 (reference) | 4.72% |  |
| 2 | Begg CB 1998 | United States | 1984-1993 | Retrospective | 503 | 126 | esophagectomy | 1-5 | NR | 17.3% | 30-day |
|  |  |  |  |  |  |  |  | 6-10 | NR | 3.88% |  |
|  |  |  |  |  |  |  |  | ≥11 | NR | 3.4% |  |
| 3 | Urbach DR 2004 | Canada | 1994-1999 | Retrospective | 613 | 477 | oesophagectomy | 0.2-8.8 | 1 (reference) | 15.55% | 30-day |
|  |  |  |  |  |  |  |  | 8.8-19 | 0.60 (0.30 to 1.20) | 10.88% |  |
| 4 | Dimick JB 2001 | United States | 1984-1999 | Retrospective | 1136 | 52 | esophagectomy | <4 | 1 (reference) | 16% | In-hospital |
|  |  |  |  |  |  |  |  | 4-15 | 0.73 (0.47–1.10) | 12.7% |  |
|  |  |  |  |  |  |  |  | ＞15 | 0.21 (0.10 – 0.42) | 2.7% |  |
| 5 | Nimptsch U 2018 | Germany | 2010-2015 | Retrospective | 22700 | 422 | complex | 1-4 | NR | 12.2% | In-hospital |
|  |  |  |  |  |  |  | esophageal | 9-11 | NR | 10.0% |  |
|  |  |  |  |  |  |  | surgery | 14-17 | NR | 10.0% |  |
|  |  |  |  |  |  |  |  | 23-32 | NR | 8.7% |  |
|  |  |  |  |  |  |  |  | 49-76 | NR | 6.8% |  |
| 6 | Kozower BD 2012 | United States | 2007 | Retrospective | 1210 | 217 | esophagectomy | 1 | 12.69 (0.54–299.72) | 12.82% | In-hospital |
|  |  |  |  |  |  |  |  | 2 | 4.09 (0.15–114.57) | 13.41% |  |
|  |  |  |  |  |  |  |  | 3 | 3.03 (0.05–201.15) | 6.35% |  |
|  |  |  |  |  |  |  |  | 4-7 | 2.77 (0.10–73.54) | 5.50% | In-hospital |
|  |  |  |  |  |  |  |  | 8-120 | 1 Reference | 2.21% |  |
| 7 | Schlottmann F 2018 | United States | 2000-2014 | Retrospective | 5235 | NR | esophagectomy | <5 | 2.17 (1.49–3.15) | NR | In-hospital |
|  |  |  |  |  |  |  |  | 5-20 | 1.62 (1.20–2.17) | NR |  |
|  |  |  |  |  |  |  |  | >20 | 1 reference | NR |  |
| 8 | Ghaferi AA 2011 | United States | 2005-2007 | Retrospective | NR | NR | esophagectomy | <1.3 | 3.70 (2.74–4.98) | 17.5% | In-hospital/30-day |
|  |  |  |  |  |  |  |  | NR | NR | 15.1% |  |
|  |  |  |  |  |  |  |  | NR | NR | 12.9% |  |
|  |  |  |  |  |  |  |  | NR | NR | 8.1% |  |
|  |  |  |  |  |  |  |  | >15 | 1 reference | 5.3% |  |
| 9 | van Lanschot JJ 2001 | Netherlands | 1993-1998 | Retrospective | 3692 | 100 | esophagectomy | ≤10 | NR | 12.1% | In-hospital |
|  |  |  |  |  |  |  |  | 11-20 | NR | 7.5% |  |
|  |  |  |  |  |  |  |  | >20 | NR | 4.9% |  |
| 10 | Finlayson EA 2003 | United States | 1995-1997 | Retrospective | 5282 | 603 | esophagectomy | <4 | NR | 15.0% | In-hospital |
|  |  |  |  |  |  |  |  | 4-9 | NR | 13.8% |  |
|  |  |  |  |  |  |  |  | >9 | NR | 6.5% |  |
| 11 | Birkmeyer JD 2002 | United States | 1994-1999 | Retrospective | 6337 | 1575 | esophagectomy | <2 | 1 reference | 20.3% | In-hospital/30-day |
|  |  |  |  |  |  |  |  | 2-4 | 0.85 (0.69–1.05) | 17.8% |  |
|  |  |  |  |  |  |  |  | 5-7 | 0.76 (0.60–0.97) | 16.2% |  |
|  |  |  |  |  |  |  |  | 8-19 | 0.51 (0.40–0.64) | 11.4% |  |
|  |  |  |  |  |  |  |  | >19 | 0.36 (0.26–0.50) | 8.4% |  |
| 12 | Fumagalli U 2013 | Italy | 2005-2011 | Retrospective | 2801 | 111 | esophagectomy, | <7.1 | 1 reference | 5.7% | 30-day |
|  |  |  |  |  |  |  | esophagectomy | 7-21 | 0.47 (0.28–0.78) | 2.6% |  |
|  |  |  |  |  |  |  | and gastrectomy | >21 | 0.36 (0.20–0.53) | 1.7% |  |
| 13 | Patti MG 1998 | United States | 1990-1994 | Retrospective | 1561 | 273 | esophagectomy | 0.2-1 | NR | 17% | In-hospital |
|  |  |  |  |  |  |  |  | 1.2-2 | NR | 19% |  |
|  |  |  |  |  |  |  |  | 2.2-4 | NR | 10% |  |
|  |  |  |  |  |  |  |  | 4.2-6 | NR | 16% |  |
|  |  |  |  |  |  |  |  | >6 | NR | 6% |  |
| 14 | Birkmeyer JD 2006 | United States | 2000-2002 | Retrospective | 6439 | 2934 | esophagectomy | Q1 | 2.34 (1.58-3.46) | 15.2% | In-hospital/30-day |
|  |  |  |  |  |  |  |  | Q2 | NR | 14.4% |  |
|  |  |  |  |  |  |  |  | Q3 | NR | 11.3% |  |
|  |  |  |  |  |  |  |  | Q4 | NR | 9.7% |  |
|  |  |  |  |  |  |  |  | Q5 | 1 reference | 7.1% |  |
| 15 | Al-Sarira AA 2007 | United Kingdom | 1997-1999 | Retrospective | 5349 | 180/167 | oesophageal | ≤9 | NR | 13.0% | In-hospital |
|  |  |  |  |  |  |  | resections: | 10-19 | NR | 13.9% |  |
|  |  |  |  |  |  |  | oesophagectomy | 20-29 | NR | 12.7% |  |
|  |  |  |  |  |  |  | or | 30-39 | NR | 9.0% |  |
|  |  |  |  |  |  |  | oesophagogastrectomy | ≥40 | NR | 6.9% |  |
|  |  | United Kingdom | 2000-2001 | Retrospective | 3260 | 152/139 | oesophageal | ≤9 | NR | 12.7% | In-hospital |
|  |  |  |  |  |  |  | resections: | 10-19 | NR | 11.6% |  |
|  |  |  |  |  |  |  |  | 20-29 | NR | 9.2% |  |
|  |  |  |  |  |  |  |  | 30-39 | NR | 10.0% |  |
|  |  |  |  |  |  |  |  | ≥40 | NR | 6.2% |  |
|  |  | United Kingdom | 2002-2003 | Retrospective | 3229 | 127/111 | oesophageal | ≤9 | NR | 11.8% | In-hospital |
|  |  |  |  |  |  |  | resections: | 10-19 | NR | 8.3% |  |
|  |  |  |  |  |  |  |  | 20-29 | NR | 6.0% |  |
|  |  |  |  |  |  |  |  | 30-39 | NR | 9.0% |  |
|  |  |  |  |  |  |  |  | ≥40 | NR | 4.5% |  |
| 16 | Funk LM 2011 | United States | 2004-2007 | Retrospective | 4498 | 874 | esophagectomy | 0-1 | 2.2(1.3-3.7) | 10.4% | In-hospital/30-day |
|  |  |  |  |  |  |  |  | 2-4 | 1.6(1.0-2.5) | 6.0% |  |
|  |  |  |  |  |  |  |  | 9-20 | 1 reference | 2.9% |  |
| 17 | Fujita H 2009 | Japan | 2001-2006 | Retrospective | 31380 | 709 | esophagectomy | 0-0.7 | 1 reference | 2% | 30-day |
|  |  |  |  |  |  |  |  | 0.8-1.5 | 0.735(0.545-0.992) | 1.5% |  |
|  |  |  |  |  |  |  |  | 1.7-3.2 | 0.621(0.456-0.847) | 1.3% |  |
|  |  |  |  |  |  |  |  | 3.3-6.5 | 0.626(0.459-0.855) | 1.3% |  |
|  |  |  |  |  |  |  |  | 6.7-13.2 | 0.327(0.221-0.483) | 0.7% |  |
|  |  |  |  |  |  |  |  | ≥13.3 | 0.307(0.181-0.518) | 0.6% |  |
|  |  | Japan | 2001-2006 | Retrospective | 31380 | 709 | esophagectomy | 0-0.7 | 1 reference | 6% | In-hospital |
|  |  |  |  |  |  |  |  | 0.8-1.5 | 0.782(0.656-0.931) | 4.7% |  |
|  |  |  |  |  |  |  |  | 1.7-3.2 | 0.698(0.584-0.835) | 4.2% |  |
|  |  |  |  |  |  |  |  | 3.3-6.5 | 0.548(0.453-0.664) | 3.2% |  |
|  |  |  |  |  |  |  |  | 6.7-13.2 | 0.370(0.297-0.461) | 2.2% |  |
|  |  |  |  |  |  |  |  | ≥13.3 | 0.288(0.210-0.394) | 1.8% |  |
| 18 | Kazui T 2007 | Japan | 2000-2004 | Retrospective | 21020 | 551 | esophageal | 1-4 | 2.27(1.54-3.33) | 6.85% | In-hospital |
|  |  |  |  |  |  |  | cancer | 5-9 | 2.21(1.53-3.21) | 5.52% |  |
|  |  |  |  |  |  |  | surgery | 10-14 | 1.82(1.22-2.70) | 4.16% |  |
|  |  |  |  |  |  |  |  | 15-19 | 1.61(0.94-2.76) | 4.02% |  |
|  |  |  |  |  |  |  |  | 20-29 | 1.20(0.73-1.98) | 3.16% |  |
|  |  |  |  |  |  |  |  | 30-39 | 0.96(0.62-1.49) | 2.43% |  |
|  |  |  |  |  |  |  |  | ≥40 | 1 reference | 2.95% |  |
| 19 | Ling HC 2006 | Taiwan | 2000-2003 | Retrospective | 6674 | 111 | esophagectomy | <19.5 | 1 reference | 6.79% | In-hospital |
|  |  |  |  |  |  |  |  | 19.5-33.8 | 0.97(0.68-1.39) | 7.60% |  |
|  |  |  |  |  |  |  |  | 34-58.8 | 0.87(0.62-1.23) | 5.31% |  |
|  |  |  |  |  |  |  |  | 59-86.5 | 0.72(0.47-0.91) | 4.22% |  |
|  |  |  |  |  |  |  |  | >86.5 | 0.65(0.43-0.97) | 5.49% |  |
| 20 | Reavis KM 2008 | United States | 2003-2007 | Retrospective | 5236 | 107 | esophagectomy | ≤5 | NR | 5.6% | In-hospital |
|  |  |  |  |  |  |  |  | 6-12 | NR | 4.7% |  |
|  |  |  |  |  |  |  |  | >12 | NR | 2.5% |  |
| 21 | Stavrou EP 2010 | Australia | 2000-2006 | Retrospective | 321 | NR | oesophagectomy | ≤1.43 | NR | 6.4% | 30-day |
|  |  |  |  |  |  |  |  | 1.4-2.9 | NR | 4.3% |  |
|  |  |  |  |  |  |  |  | ≥2.9 | NR | 2.6% |  |
| 22 | Suzuki H 2010 | Japan | 2006-2007 | Retrospective | 11503 | 728 | esophagectomy | 1-5 | NR | 4.5% | In-hospital/30-day |
|  |  |  |  |  |  |  |  | 6-20 | NR | 4.6% |  |
|  |  |  |  |  |  |  |  | 21-50 | NR | 1.7% |  |
|  |  |  |  |  |  |  |  | >51 | NR | 1.8% |  |
| 23 | Wouters MW 2009 | Netherlands | 1990-2004 | Retrospective | 214 | 11 | esophagectomy | <10 | NR | 6.3% | In-hospital |
|  |  |  |  |  |  |  |  | ≥10 | NR | 2.9% |  |
| 24 | Kuo EY 2001 | United States | 1992-2000 | Retrospective | 1193 | 64 | esophagectomy | high(average=28) | 1 reference | 2.5% | In-hospital |
|  |  |  |  |  |  |  |  | low(average=1.1） | 4.3 (2.3–7.7) | 9.2% |  |
| 25 | Sheetz KH 2019 | United States | 2005-2016 | Retrospective | 29812 | NR | esophagectomy | 2-4 | 1 reference | 6.6% | 30-day |
|  |  |  |  |  |  |  |  | 57-98 | 0.59(0.41-0.85) | 4.2% |  |
| 26 | Fischer C 2017 | United Kingdom | 2011-2013 | Retrospective | 4868 | 42 | esophagectomy | 0-49 | 0.94(0.91-0.98) | 3.0% | 30-day |
|  |  |  |  |  |  |  |  | 50-65 | NR | 3.1% |  |
|  |  |  |  |  |  |  |  | 66-91 | NR | 1.7% |  |
|  |  |  |  |  |  |  |  | 92-148 | 0.96(0.93-0.98) | 1.3% |  |
| 27 | Dimick JB 2005 | United States | 1998-1999 | Retrospective | 1946 | NR | esophageal cancer | <5 | NR | 17.1% | In-hospital/30-day |
|  |  |  |  |  |  |  | resection | 5-12 | NR | 13.7% |  |
|  |  |  |  |  |  |  |  | >12 | NR | 10.6% |  |
| 28 | McCulloch P 2003 | United Kingdom | 1999-2002 | Retrospective | 955 | 32 | oesophagogastric | 0-10 | 1 reference | 16.7% | In-hospital |
|  |  |  |  |  |  |  |  | 11-20 | 0.50(0.24-1.05) | 12.7% |  |
|  |  |  |  |  |  |  |  | 21-39 | 0.49(0.24-0.97) | 10.3% |  |
| 29 | Wenner G 2005 | Sweden | 1987-1996 | Retrospective | 1429 | 74 | Resection + | <5 | NR | 11.2% | NR |
|  |  |  |  |  |  |  | oesophago | 5-15 | NR | 6.1% |  |
|  |  |  |  |  |  |  | gastrostomy | >15 | NR | 2.9% |  |
|  |  | Sweden | 1987-1996 | Retrospective | 1429 | 74 | Total gastrectomy | <5 | NR | 9.1% | NR |
|  |  |  |  |  |  |  | +oesophagojejunostomy | 5-15 | NR | 4.0% |  |
|  |  |  |  |  |  |  |  | >15 | NR | 3.0% |  |
| 30 | Gillison EW 2002 | United Kingdom | 1992-1996 | Retrospective | 1125 | 19 | resection of | <2 | NR | 23.8% | 30-day |
|  |  |  |  |  |  |  | carcinoma of | 5-8 | NR | 8.4% |  |
|  |  |  |  |  |  |  | the oesophagus | 11-19 | NR | 10.7% |  |
|  |  |  |  |  |  |  | and cardia | >20 | NR | 10.2% |  |
| 31 | Ulrich G 2017 | Switzerland | 1999-2012 | Retrospective | 1487 | NR | oesophagectomy | 1-10 | 1 reference | 6.30% | In-hospital |
|  |  |  |  |  |  |  |  | >10 | 0.5(0.22-1.18) | 3.30% |  |
| 32 | Gasper WJ 2009 | United States | 1990-1994 | Retrospective | 1561 | 273 | esophageal resection | <6 | NR | 17% | In-hospital/30-day |
|  |  |  |  |  |  |  |  | 6-10 | NR | 19% |  |
|  |  |  |  |  |  |  |  | 11-20 | NR | 10% |  |
|  |  |  |  |  |  |  |  | 21-30 | NR | 16% |  |
|  |  |  |  |  |  |  |  | >30 | NR | 6% |  |
|  |  | United States | 1995-1999 | Retrospective | 1194 | 224 | esophageal resection | <6 | 1.95(1.03-3.69) | 8.4% | In-hospital/30-day |
|  |  |  |  |  |  |  |  | 6-10 | 1.01(0.50-2.06) | 5.6% |  |
|  |  |  |  |  |  |  |  | 11-20 | 1.59(0.84-3.03) | 6.8% |  |
|  |  |  |  |  |  |  |  | 21-30 | 1.29(0.58-2.86) | 5.1% |  |
|  |  |  |  |  |  |  |  | >30 | 1 reference | 4.9% |  |
|  |  | United States | 2000-2004 | Retrospective | 1210 | 183 | esophageal resection | <6 | 1.65(1.01-2.69) | 7.4% | In-hospital/30-day |
|  |  |  |  |  |  |  |  | 6-10 | 1.45(0.78-2.68) | 4.9% |  |
|  |  |  |  |  |  |  |  | 11-20 | 1.19(0.57-2.47) | 6.0% |  |
|  |  |  |  |  |  |  |  | 21-30 | 0.94(0.45-1.98) | 4.5% |  |
|  |  |  |  |  |  |  |  | >30 | 1 reference | 4.4% |  |
| 33 | Urbach DR 2005 | Canada | 1994-1999 | Retrospective | 4868 | 58 | esophagectomy | 0.2-2.1 | NR | 18.59% | 30-day |
|  |  |  |  |  |  |  |  | 2.2-7.0 | NR | 10.59% |  |
|  |  |  |  |  |  |  |  | 7.1-12.0 | NR | 14.57% |  |
|  |  |  |  |  |  |  |  | 12.1-14.4 | NR | 9.56% |  |
| 34 | Rouvela L 2007 | Sweden | 1987-2000 | Retrospective | 1199 | NR | esophagectomy | <10 | NR | 8.80% | 30-day |
|  |  |  |  |  |  |  |  | ≥10 | NR | 4.30% |  |
| 35 | Dimick JB 2003 | United States | 1995-1999 | Retrospective | 3023 | NR | esophageal resection | <3 | 2.9(1.7-4.9) | 11.8% | In-hospital |
|  |  |  |  |  |  |  |  | 3-5 | 2.4(1.4-4.3) | 10.2% |  |
|  |  |  |  |  |  |  |  | 6-16 | NR | 6.4% |  |
|  |  |  |  |  |  |  |  | >16 | 1 reference | 3.7% |  |
| 36 | Dimick JB 2005 | United States | 1988-1991 | Retrospective | 1963 | NR | esophageal resection | ≤6 | NR | 15.3% | In-hospital |
|  |  |  |  |  |  |  |  | >6 | NR | 11.0% |  |
|  |  | United States | 1992-1996 | Retrospective | 3663 | NR | esophageal resection | ≤6 | NR | 14.7% | In-hospital |
|  |  |  |  |  |  |  |  | >6 | NR | 7.3% |  |
|  |  | United States | 1997-2000 | Retrospective | 3031 | NR | esophageal resection | ≤6 | NR | 14.5% | In-hospital |
|  |  |  |  |  |  |  |  | >6 | NR | 7.4% |  |
| 37 | Swisher SG 2000 | United States | 1994-1996 | Retrospective | 340 | 25 | esophagectomy | <5 | 3.97(1.14-13.84) | 12.2% | In-hospital |
|  |  |  |  |  |  |  |  | ≥5 | 1 (reference) | 3.0% |  |
| 38 | Munasinghe A 2015 | United States | 2005-2010 | Retrospective | 5858 | 775 | esophagectomy | <27 | NR | 6.55% | 30-day |
|  |  |  |  |  |  |  |  | 27-111 | 0.98(0.97-0.98) | 2.10% |  |
|  |  | England | 2005-2010 | Retrospective | 7433 | 66 | esophagectomy | <26 | NR | 5.00% | 30-day |
|  |  |  |  |  |  |  |  | 26.4-81.6 | 0.99(0.98-1.00) | 3.50% |  |
| 39 | Nimptsch U 2017 | Germany | 2009-2014 | Retrospective | 18208 | 375 | complex oesophageal | 1-4 | 1 reference | 10.50% | In-hospital |
|  |  |  |  |  |  |  | surgery for carcinoma | 7-10 | 0.81(0.68-0.96) | NR |  |
|  |  |  |  |  |  |  |  | 12-16 | 0.85(0.72-1.01) | NR |  |
|  |  |  |  |  |  |  |  | 21-29 | 0.67(0.56-0.82) | NR |  |
|  |  |  |  |  |  |  |  | 42-67 | 0.47(0.36-0.58) | 5.80% |  |
| 40 | Steyerberg EW 2006 | Netherlands | 1991-1996 | Retrospective | 1327 | NR | esophagectomy | ≤1 | 1 reference | 14% | 30-day |
|  |  |  |  |  |  |  |  | 1.1-2.5 | 0.80(0.52-1.2) | 12% |  |
|  |  |  |  |  |  |  |  | ≥2.6 | 0.59(0.39-0.90) | 8% |  |
|  |  | Netherlands | 1997-1999 | Retrospective | 714 | NR | esophagectomy | ≤1 | 1 reference | 14% | 30-day |
|  |  |  |  |  |  |  |  | 1.1-2.5 | 1.5(0.82-2.6) | 18% |  |
|  |  |  |  |  |  |  |  | ≥2.6 | 0.36(0.19-0.69) | 5% |  |
| 41 | Nimptsch U 2016 | Germany | 2006-2013 | Retrospective | 28931 | 423 | complex interventions | 1-5 | 1 reference | 12.7% | In-hospital |
|  |  |  |  |  |  |  |  | 11-22 | 0.70(0.62-0.80) | 9.0% |  |
| 42 | Finley CJ 2011 | Canada | 1998-2007 | Retrospective | 6985 | NR | esophagectomy | <6 | NR | 9.80% | In-hospital |
|  |  |  |  |  |  |  |  | 7-19 | NR | NR |  |
|  |  |  |  |  |  |  |  | >20 | NR | 4.8% |  |
| 43 | Leigh Y 2009 | United Kingdom | 1998-2003 | Retrospective | 9034 | NR | oesophagectomy | <20 | 1.43(1.18-1.74) | 6.3% | 30-day |
|  |  |  |  |  |  |  |  | >20 | 1 reference | 9.6% |  |
| 44 | Wouters MW 2008 | Netherlands | 1990-1999 | Retrospective | 903 | 11 | esophageal resection | ≤7 | 3.05(1.82-5.11) | 13% | In-hospital |
|  |  |  |  |  |  |  |  | >7 | 1 reference | 5% |  |
| 45 | Ra J 2008 | United States | 1997-2003 | Retrospective | 1172 | 361 | NR | <0.67 | 1.81(1.18-2.78) | 16.3% | In-hospital/30-day |
|  |  |  |  |  |  |  |  | 0.68-2.33 | 1.68(1.07-2.66) | 14.8% |  |
|  |  |  |  |  |  |  |  | >2.33 | 1 reference | 9.7% |  |
| 46 | Allaredd V 2007 | United States | 2000-2003 | Retrospective | 2473 | 717 | esophagectomy | ＜13 | 1.98 (1.28–3.07) | NR | In-hospital |
|  |  |  |  |  |  |  |  | ≥13 | 1 (reference) | NR |  |
| 47 | Simunovic M 2006 | Canada | 1990-2000 | Retrospective | 629 | 68 | esophageal resection | ≤1.17 | 0.9 (0.3–2.5) [0.83] | 12.9% | In-hospital |
|  |  |  |  |  |  |  |  | 1.33-3.17 | 0.8 (0.3–1.9) [0.59] | 11.5% |  |
|  |  |  |  |  |  |  |  | 3.33-7.17 | 0.5 (0.2–1.2) [0.10] | 5.8% |  |
|  |  |  |  |  |  |  |  | ≥7.33 | 1 (reference) | 11.8% |  |
| 48 | Dimick JB 2003 | United States | 1994-1998 | Retrospective | 366 | 52 | esophageal resection | ＜34 | 5.7(2.0-16.0) | 15.4% | In-hospital |
|  |  |  |  |  |  |  |  | ≥34 | 1 reference | 2.5% |  |
| 49 | Urbach DR 2003 | Canada | 1994-1999 | Retrospective | 613 | 47 | esophagectomy | 2.8 | NR | 18.6% | 30-day |
|  |  |  |  |  |  |  |  | 8.8 | NR | 12.6% |  |
|  |  |  |  |  |  |  |  | 16.6 | NR | 12.0% |  |
|  |  |  |  |  |  |  |  | 19 | NR | 10.2% |  |
| 50 | Dikken JL2013 | Netherlands, | 2004-2009 | Retrospective | 10854 | NR | esophagectomy | 1-10 | 1 (reference) | 7.20% | 30-day |
|  |  | Sweden, |  |  |  |  |  | 11-20 | 0.82 (0.61-1.11) | NR |  |
|  |  | Denmark and |  |  |  |  |  | 21-30 | 0.68 (0.50-0.93) | NR |  |
|  |  | England |  |  |  |  |  | 31-40 | 0.58 (0.39-0.85) | NR |  |
|  |  |  |  |  |  |  |  | ≥41 | 0.55 (0.42-0.72) | 4.30% |  |
| 51 | Rodgers M 2007 | United States | 1988-2000 | Retrospective | 3243 | NR | esophagectomy | 1-4 | NR | 11.37% | In-hospital |
|  |  |  |  |  |  |  |  | 5-9 | NR | 7.78% |  |
|  |  |  |  |  |  |  |  | >9 | NR | 11.55% |  |
| 52 | Markar S 2015 | France | 2000-2010 | Retrospective | 2944 | 30 | esophagectomy | ≤80 | 2.62(1.77-3.87) | 10.50% | 30-day |
|  |  |  |  |  |  |  |  | >80 | 1 rererence | 3.00% |  |
| 53 | Voeten DM 2021 | Netherlands | 2016-2019 | Retrospective | 3135 | 16 | esophagectomy | <40 | 1 reference | 2.8% | In-hospital/30-day |
|  |  |  |  |  |  |  |  | >40 | 0.93(0.57-1.58) | 2.6% |  |
|  |  |  |  |  |  |  |  | <53 | 1 reference | 2.6% |  |
|  |  |  |  |  |  |  |  | >53 | 1.02(0.67-1.56) | 2.8% |  |
| 54 | Kennedy GT 2018 | United States | 2004-2013 | Prospective | 9270 | NR | esophagectomy | <7 | 1 (reference) | 8.9% | 30-day |
|  |  |  |  |  |  |  |  | 7-22 | NR | NR |  |
|  |  |  |  |  |  |  |  | 23-87 | 0.46(0.23-0.88) | NR |  |
|  |  |  |  |  |  |  |  | >87 | 0.047(0.007-0.40) | 3.6% |  |
| 55 | Sakata R 2012 | Japan | 2005-2009 | Retrospective | 24224 | 493 | esophageal cancer | 1-4 | 3.23(2.02-5.15) | 2.41% | 30-day |
|  |  |  |  |  |  |  | surgery | 5-9 | 3.59(2.28-5.65) | 2.03% |  |
|  |  |  |  |  |  |  |  | 10-14 | 2.31(1.37-3.90） | 1.30% |  |
|  |  |  |  |  |  |  |  | 15-19 | 2.82(1.37-5.81) | 1.56% |  |
|  |  |  |  |  |  |  |  | 20-29 | 1.65(0.91-3.00) | 1.01% |  |
|  |  |  |  |  |  |  |  | 30-39 | 1.33(0.77-2.29) | 0.79% |  |
|  |  |  |  |  |  |  |  | >40 | 1 (reference) | 0.60% |  |
| 56 | Allareddy V 2010 | United States | 2000-2003 | Retrospective | 2473 | 555 | esophagectomy | <13 | 1 reference | 9.68% | In-hospital |
|  |  |  |  |  |  |  |  | ≥13 | 0.53(0.35-0.82) | 4.42% |  |

**Supplemental Table 3** Risk of postoperative mortality and covariates in adjusted factors.

| Study | Period | Hospital volume (cases/year) | Odds ratio (95% CI) | Adjusted factors |
| --- | --- | --- | --- | --- |
| Reames BN 2014 | 2000-2001 | <2 | 2.25 (1.57 - 3.23) | Age, sex, race (black or non-black) and their interactions, urgency or emergency of the admission, the presence of coexisting conditions, and social economic status. |
|  |  | >12 | 1 reference |  |
|  | 2002-2003 | <2 | 1.92 (1.36 - 2.70) |  |
|  |  | >12 | 1 reference |  |
|  | 2004-2005 | <2 | 3.18 (2.41 - 4.18) |  |
|  |  | >17 | 1 reference |  |
|  | 2006-2007 | <2 | 2.41 (1.66 - 3.52) |  |
|  |  | >17 | 1 reference |  |
|  | 2008-2009 | <3 | 3.68 (2.66 - 5.11) |  |
|  |  | >18 | 1 reference |  |
| Begg CB 1998 | 1984-1993 | 1--5 | NR | Comorbidity, patient age, and cancer stage. |
|  |  | 6--10 | NR |  |
|  |  | ≥11 | NR |  |
| Urbach DR 2004 | 1994-1999 | 0.2-8.8 | 1 reference | Age, sex, and comorbidity. |
|  |  | 8.8-19 | 0.60 (0.30 to 1.20) |  |
| Dimick JB 2001 | 1984-1999 | <4 | 1 (reference) | Demographics (age, sex, and race), comorbid disease, and severity of illness. |
|  |  | 4-15 | 0.73 (0.47–1.10) |  |
|  |  | ＞15 | 0.21 (0.10 – 0.42) |  |
| Nimptsch U 2018 | 2010-2015 | 1-4 | NR | Age, sex, calendar year of treatment, certain comorbidities and underlying disease. |
|  |  | 9-11 | NR |  |
|  |  | 14-17 | NR |  |
|  |  | 23-32 | NR |  |
|  |  | 49-76 | NR |  |
| Kozower BD 2012 | 2007 | 1 | 12.69 (0.54–299.72) | Age, gender, and comorbid disease. |
|  |  | 2 | 4.09 (0.15–114.57) |  |
|  |  | 3 | 3.03 (0.05–201.15) |  |
|  |  | 4-7 | 2.77 (0.10–73.54) |  |
|  |  | 8-120 | 1 Reference |  |
| Schlottmann F 2018 | 2000-2014 | <5 | 2.17 (1.49–3.15) | Admit year, age (modeled as a restricted cubic spline), sex, race/ethnicity, insurance type, income, comorbidities, hospital size, location/teaching status, and region. |
|  |  | 5-20 | 1.62 (1.20–2.17) |  |
|  |  | >20 | 1 reference |  |
| Ghaferi AA 2011 | 2005-2007 | <1.3 | 3.70 (2.74–4.98) | Age, sex, race, urgency of operation, and comorbidities. |
|  |  | NR | NR |  |
|  |  | NR | NR |  |
|  |  | NR | NR |  |
|  |  | >15 | 1 reference |  |
| van Lanschot JJ 2001 | 1993-1998 | ≤10 | NR | NR. |
|  |  | 11-20 | NR |  |
|  |  | >20 | NR |  |
| Finlayson EA 2003 | 1995-1997 | <4 | NR | Age , sex ,race , year of procedure, acuity of the admission, patient comorbidities, and median social security income. |
|  |  | 4-9 | NR |  |
|  |  | >9 | NR |  |
| Birkmeyer JD 2002 | 1994-1999 | <2 | 1 reference | Aage, sex, race, year of procedure, social security income, urgency of admission, and Charlson score, |
|  |  | 2-4 | 0.85 (0.69–1.05) | a measure of coexisting conditions. |
|  |  | 5-7 | 0.76 (0.60–0.97) |  |
|  |  | 8-19 | 0.51 (0.40–0.64) |  |
|  |  | >19 | 0.36 (0.26–0.50) |  |
| Fumagalli U 2013 | 2005-2011 | <7.1 | 1 reference | Aage, sex, and comorbidity index. |
|  |  | 7-21 | 0.47 (0.28–0.78) |  |
|  |  | >21 | 0.36 (0.20–0.53) |  |
| Patti MG 1998 | 1990-1994 | 0.2-1 | NR | Year of operation, age, sex, race, payer source, location of tumor, incresing no.of secondary diagnoses. |
|  |  | 1.2-2 | NR |  |
|  |  | 2.2-4 | NR |  |
|  |  | 4.2-6 | NR |  |
|  |  | >6 | NR |  |
| Birkmeyer JD 2006 | 2000-2002 | Q1 | 2.34 (1.58-3.46) | Adjusted for patient and hospital characteristics and all measurable processes of care. |
|  |  | Q2 | NR | Patient characteristics included age group (5-year intervals), sex, race (black, nonblack), cancer type, year of procedures, and |
|  |  | Q3 | NR | admission acuity (elective, urgent/emergent). Hospital characteristics, including ownership (not-for-profit and for-profit) and |
|  |  | Q4 | NR | teaching status. |
|  |  | Q5 | 1 reference |  |
| Al-Sarira AA 2007 | 1997-1999 | ≤9 | NR | NR. |
|  |  | 10-19 | NR |  |
|  |  | 20-29 | NR |  |
|  |  | 30-39 | NR |  |
|  |  | ≥40 | NR |  |
|  | 2000-2001 | ≤9 | NR | NR. |
|  |  | 10-19 | NR |  |
|  |  | 20-29 | NR |  |
|  |  | 30-39 | NR |  |
|  |  | ≥40 | NR |  |
|  | 2002-2003 | ≤9 | NR | NR. |
|  |  | 10-19 | NR |  |
|  |  | 20-29 | NR |  |
|  |  | 30-39 | NR |  |
|  |  | ≥40 | NR |  |
| Funk LM 2011 | 2004-2007 | 0-1 | 2.2 (1.3-3.7) | Patient characteristics = gender + age + race + admission type + Charlson score>=3, and Systems characteristics = high nurse ratio |
|  |  | 2-4 | 1.6 (1.0-2.5) | + complex medical oncology services + lung transplantation services + bariatric surgery services + PET scanner. |
|  |  | 9-20 | 1 reference |  |
| Fujita H 2009 | 2001-2006 | 0-0.7 | 1 reference | NR. |
|  |  | 0.8-1.5 | 0.735 (0.545-0.992) |  |
|  |  | 1.7-3.2 | 0.621 (0.456-0.847) |  |
|  |  | 3.3-6.5 | 0.626 (0.459-0.855) |  |
|  |  | 6.7-13.2 | 0.327 (0.221-0.483) |  |
|  |  | ≥13.3 | 0.307 (0.181-0.518) |  |
|  |  | 0-0.7 | 1 reference | NR. |
|  |  | 0.8-1.5 | 0.782 (0.656-0.931) |  |
|  |  | 1.7-3.2 | 0.698 (0.584-0.835) |  |
|  |  | 3.3-6.5 | 0.548 (0.453-0.664) |  |
|  |  | 6.7-13.2 | 0.370 (0.297-0.461) |  |
| Kazui T 2007 | 2000-2004 | 1-4 | 2.27 (1.54-3.33) | NR. |
|  |  | 5-9 | 2.21 (1.53-3.21) |  |
|  |  | 10-14 | 1.82 (1.22-2.70) |  |
|  |  | 15-19 | 1.61 (0.94-2.76) |  |
|  |  | 20-29 | 1.20 (0.73-1.98) |  |
|  |  | 30-39 | 0.96 (0.62-1.49) |  |
|  |  | ≥40 | 1 reference |  |
| Ling HC 2006 | 2000-2003 | <19.5 | 1 reference | NR. |
|  |  | 19.5-33.8 | 0.97 (0.68-1.39) |  |
|  |  | 34-58.8 | 0.87 (0.62-1.23) |  |
|  |  | 59-86.5 | 0.72 (0.47-0.91) |  |
|  |  | >86.5 | 0.65 (0.43-0.97) |  |
| Reavis KM 2008 | 2003-2007 | ≤5 | NR | NR. |
|  |  | 6-12 | NR |  |
|  |  | >12 | NR |  |
| Stavrou EP 2010 | 2000-2006 | ≤1.43 | NR | NR. |
|  |  | 1.4-2.9 | NR |  |
|  |  | ≥2.9 | NR |  |
| Suzuki H 2010 | 2006-2007 | 1-5 | NR | NR. |
|  |  | 6-20 | NR |  |
|  |  | 21-50 | NR |  |
|  |  | >51 | NR |  |
| Wouters MW 2009 | 1990-2004 | <10 | NR | NR. |
|  |  | ≥10 | NR |  |
| Kuo EY 2001 | 1992-2000 | High (average=28) | 1 reference | Age, race, comorbidity score, urgency of admission, source of admission, year, payer type, and residence. |
|  |  | Low (average=1.1） | 4.3 (2.3–7.7) |  |
| Sheetz KH 2019 | 2005-2016 | 2-4 | 1 reference | NR. |
|  |  | 57-98 | 0.59 (0.41-0.85) |  |
| Fischer C 2017 | 2011-2013 | 0-49 | 0.94 (0.91-0.98) | Comorbidity count, age, ASA score, ECOG (WHO) performance status, Tstage, N stage, cancer location, surgeon volume. |
|  |  | 50-65 | NR |  |
|  |  | 66-91 | NR |  |
|  |  | 92-148 | 0.96 (0.93-0.98) |  |
| Dimick JB 2005 | 1998-1999 | <5 | NR | Age, gender, race, urgent, emergent, charlson score, lowest income group. |
|  |  | 5-12 | NR |  |
|  |  | >12 | NR |  |
| McCulloch P 2003 | 1999-2002 | 0-10 | 1 reference | NR. |
|  |  | 11-20 | 0.50 (0.24-1.05) |  |
|  |  | 21-39 | 0.49 (0.24-0.97) |  |
| Wenner G 2005 | 1987-1996 | <5 | NR | NR. |
|  |  | 5-15 | NR |  |
|  |  | >15 | NR |  |
|  | 1987-1996 | <5 | NR | NR. |
|  |  | 5-15 | NR |  |
|  |  | >15 | NR |  |
| Gillison EW 2002 | 1992-1996 | <2 | NR | NR. |
|  |  | 5-8 | NR |  |
|  |  | 11-19 | NR |  |
|  |  | >20 | NR |  |
| Ulrich G 2017 | 1999-2012 | 1-10 | 1 reference | Potential baseline confounding (age, gender, Swiss nationality, operation, insurance, year). |
|  |  | >10 | 0.5 (0.22-1.18) |  |
| Gasper WJ 2009 | 1990-1994 | <6 | NR | Age, gender, race, no.secondary diagoses, tumor site, payer source. |
|  |  | 6-10 | NR |  |
|  |  | 11-20 | NR |  |
|  |  | 21-30 | NR |  |
|  |  | >30 | NR |  |
|  | 1995-1999 | <6 | 1.95 (1.03-3.69) |  |
|  |  | 6-10 | 1.01 (0.50-2.06) |  |
|  |  | 11-20 | 1.59 (0.84-3.03) |  |
|  |  | 21-30 | 1.29 (0.58-2.86) |  |
|  |  | >30 | 1 reference |  |
|  | 2000-2004 | <6 | 1.65 (1.01-2.69) |  |
|  |  | 6-10 | 1.45 (0.78-2.68) |  |
|  |  | 11-20 | 1.19 (0.57-2.47) |  |
|  |  | 21-30 | 0.94 (0.45-1.98) |  |
|  |  | >30 | 1 reference |  |
| Urbach DR 2005 | 1994-1999 | 0.2-2.1 | NR | Patient age, sex, and comorbidity score. |
|  |  | 2.2-7.0 | NR |  |
|  |  | 7.1-12.0 | NR |  |
|  |  | 12.1-14.4 | NR |  |
| Rouvela L 2007 | 1987-2000 | <10 | NR | NR. |
|  |  | ≥10 | NR |  |
| Dimick JB 2003 | 1995-1999 | <3 | 2.9 (1.7-4.9) | Age, gender, race, urgent admission, emergent admission, malignacy, metastases, COPD, diabetes, history of MI, PVOD, liver |
|  |  | 3-5 | 2.4 (1.4-4.3) | disease. |
|  |  | 6-16 | NR |  |
| Dimick JB 2005 | 1988-1991 | ≤6 | NR | Age, gender, race, and coexisting diseases. |
|  |  | >6 | NR |  |
|  | 1992-1996 | ≤6 | NR |  |
|  |  | >6 | NR |  |
|  | 1997-2000 | ≤6 | NR |  |
|  |  | >6 | NR |  |
| Swisher SG 2000 | 1994-1996 | <5 | 3.97 (1.14-13.84) | Sex, age, location of tumor, type of procedure, operative mortality, complications of care,length of stay, and hospital charges. |
|  |  | ≥5 | 1 (reference) |  |
| Munasinghe A 2015 | 2005-2010 | <27 | NR | NR. |
|  |  | 27-111 | 0.98 (0.97-0.98) |  |
|  | 2005-2010 | <26 | NR |  |
|  |  | 26.4-81.6 | 0.99 (0.98-1.00) |  |
| Nimptsch U 2017 | 2009-2014 | 1-4 | 1 reference | Calendar year of treatment, age, sex, comorbidity, specific risk factor. |
|  |  | 7-10 | 0.81 (0.68-0.96) |  |
|  |  | 12-16 | 0.85 (0.72-1.01) |  |
|  |  | 21-29 | 0.67 (0.56-0.82) |  |
|  |  | 42-67 | 0.47 (0.36-0.58) |  |
| Steyerberg EW 2006 | 1991-1996 | ≤1 | 1 reference | Age, comorbidity (cardiac, pulmonary, renal, hepatic, and diabetes), preoperative radiotherapy or combined chemoradiotherapy. |
|  |  | 1.1-2.5 | 0.80 (0.52-1.2) |  |
|  |  | ≥2.6 | 0.59 (0.39-0.90) |  |
|  | 1997-1999 | ≤1 | 1 reference |  |
|  |  | 1.1-2.5 | 1.5 (0.82-2.6) |  |
|  |  | ≥2.6 | 0.36 (0.19-0.69) |  |
| Nimptsch U 2016 | 2006-2013 | 1-5 | 1 reference | Age, gender and the calendar year of treatment, certain diseases (as a main or secondary diagnosis). |
|  |  | 11-22 | 0.70 (0.62-0.80) |  |
| Finley CJ 2011 | 1998-2007 | <6 | NR | Age, sex, Charlson comorbidity index, and year of esophagectomy. |
|  |  | 7-19 | NR |  |
|  |  | >20 | NR |  |
| Leigh Y 2009 | 1998-2003 | <20 | 1.43 (1.18-1.74) | Age, sex and socio-economic deprivation. |
|  |  | >20 | 1 reference |  |
| Wouters MW 2008 | 1990-1999 | ≤7 | 3.05 (1.82-5.11) | Age,gender,histology,tumor localization,stage,anastomoses,surgical approach,(Neo)-adjuvant treatment and comorbidity. |
|  |  | >7 | 1 reference |  |
| Ra J 2008 | 1997-2003 | <0.67 | 1.81 (1.18-2.78) | Patient age, race, marital status, sex, tumor stage, Charlson score. |
|  |  | 0.68-2.33 | 1.68 (1.07-2.66) |  |
|  |  | >2.33 | 1 reference |  |
| Allaredd V 2007 | 2000-2003 | ＜13 | 1.98 (1.28–3.07) | Sex ,type of admission,age,hospital volume，hospital bed size,hospital teaching status ,in-hospital mortality,Charlson comorbid |
|  |  | ≥13 | 1 (reference) | severity index. |
| Simunovic M 2006 | 1990-2000 | ≤1.17 | 0.9 (0.3–2.5) [0.83] | Age, sex, comorbidity score,place of residence (rural v. urban)and socioeconomic status (high-income, medium-income or low- |
|  |  | 1.33-3.17 | 0.8 (0.3–1.9) [0.59] | income level). |
|  |  | 3.33-7.17 | 0.5 (0.2–1.2) [0.10] |  |
|  |  | ≥7.33 | 1 (reference) |  |
| Dimick JB 2003 | 1994-1998 | ＜34 | 5.7 (2.0-16.0) | Age, sex, race, nature of admission, operating physician, vital status at discharge, total hospital, and |
|  |  | ≥34 | 1 reference | intensive care unit length of stay. |
| Urbach DR 2003 | 1994-1999 | 2.8 | NR | Age,sex and comorbidity. |
|  |  | 8.8 | NR |  |
|  |  | 16.6 | NR |  |
|  |  | 19 | NR |  |
| Dikken JL2013 | 2004-2009 | 1-10 | 1 (reference) | Sex, age,histology,TNM stage. |
|  |  | 11-20 | 0.82 (0.61-1.11) |  |
|  |  | 21-30 | 0.68 (0.50-0.93) |  |
|  |  | 31-40 | 0.58 (0.39-0.85) |  |
|  |  | ≥41 | 0.55 (0.42-0.72) |  |
| Rodgers M 2007 | 1988-2000 | 1-4 | NR | Comorbidity, age (＞65 years), female sex, race, and surgeon volume. |
|  |  | 5-9 | NR |  |
|  |  | >9 | NR |  |
| Markar S 2015 | 2000-2010 | ≤80 | 2.62 (1.77-3.87) | Age, sex, ASA score, Tumor location, TNM stage, Srugical technique, Neoadjuvant, comorbidity. |
|  |  | >80 | 1 rererence |  |
| Voeten DM 2021 | 2016-2019 | <40 | 1 reference | Corrected for: sex, age, preoperative weight loss, BMI, Charlson Comorbidity Index, ASA score, previous esophageal or gastric |
|  |  | >40 | 0.93 (0.57-1.58) | surgery, tumor location, histology, clinical tumor stage, clinical node stage, and salvage surgery. |
|  |  | <53 | 1 reference |  |
|  |  | >53 | 1.02 (0.67-1.56) |  |
| Kennedy GT 2018 | 2004-2013 | <7 | 1 (reference) | Age, race, income, insurance, walraven score, year, region, area wage index. |
|  |  | 7-22 | NR |  |
|  |  | 23-87 | 0.46 (0.23-0.88) |  |
|  |  | >87 | 0.047 (0.007-0.40) |  |
| Sakata R 2012 | 2005-2009 | 1-4 | 3.23 (2.02-5.15) | EB estimates. |
|  |  | 5-9 | 3.59 (2.28-5.65) |  |
|  |  | 10-14 | 2.31 (1.37-3.90） |  |
|  |  | 15-19 | 2.82 (1.37-5.81) |  |
|  |  | 20-29 | 1.65 (0.91-3.00) |  |
|  |  | 30-39 | 1.33 (0.77-2.29) |  |
|  |  | >40 | 1 (reference) |  |
| Allareddy V 2010 | 2000-2003 | <13 | 1 reference | Hospital volumes (high vs. low reference), confounding effects of age, sex, primary diagnosis, extent/type of primary procedure, |
|  |  | ≥13 | 0.53 (0.35-0.82) | co-morbid severity index, year of procedure, hospital teaching status, and hospital bed size are adjusted in the analyses. |

**Supplemental Table 4** Quality assessments of included studies by Newcastle-Ottawa Scale.

| Study | Selection | | | | Comparability | Outcome | | | Total |
| --- | --- | --- | --- | --- | --- | --- | --- | --- | --- |
|  | Exposed | Nonexposed | Ascertainment | Outcome |  | Assessment | Length of | Adequacy of |  |
|  | cohort | cohort | of exposure | of interest |  | of outcome | follow-up | follow-up | score |
| Reames BN 2014 | 1 | 1 | 1 | 1 | 2 | 1 | 1 | 1 | 9 |
| Begg CB 1998 | 1 | 1 | 1 | 1 | 1 | 1 | 1 | 1 | 8 |
| Urbach DR 2004 | 1 | 1 | 1 | 1 | 1 | 1 | 1 | 1 | 8 |
| Dimick JB 2001 | 1 | 1 | 1 | 1 | 0 | 1 | 1 | 1 | 7 |
| Nimptsch U 2018 | 1 | 1 | 1 | 1 | 2 | 1 | 1 | 1 | 9 |
| Kozower BD 2012 | 1 | 1 | 1 | 1 | 1 | 1 | 1 | 1 | 8 |
| Schlottmann F 2018 | 1 | 1 | 1 | 1 | 2 | 1 | 1 | 1 | 9 |
| Ghaferi AA 2011 | 1 | 1 | 1 | 1 | 0 | 1 | 1 | 1 | 7 |
| van Lanschot JJ 2001 | 1 | 1 | 1 | 1 | 2 | 1 | 1 | 1 | 9 |
| Finlayson EA 2003 | 1 | 1 | 1 | 1 | 2 | 1 | 1 | 1 | 9 |
| Birkmeyer JD 2002 | 1 | 1 | 1 | 1 | 2 | 1 | 1 | 1 | 9 |
| Fumagalli U 2013 | 1 | 1 | 1 | 1 | 2 | 1 | 1 | 1 | 9 |
| Patti MG 1998 | 1 | 1 | 1 | 1 | 2 | 1 | 1 | 1 | 9 |
| Birkmeyer JD 2006 | 1 | 1 | 1 | 1 | 2 | 1 | 1 | 1 | 9 |
| Al-Sarira AA 2007 | 1 | 1 | 1 | 1 | 2 | 1 | 1 | 1 | 9 |
| Funk LM 2011 | 1 | 1 | 1 | 1 | 2 | 1 | 1 | 1 | 9 |
| Fujita H 2009 | 1 | 1 | 1 | 1 | 2 | 1 | 1 | 1 | 9 |
| Kazui T 2007 | 1 | 1 | 1 | 1 | 2 | 1 | 1 | 1 | 9 |
| Ling HC 2006 | 1 | 1 | 1 | 1 | 2 | 1 | 1 | 1 | 9 |
| Reavis KM 2008 | 1 | 1 | 1 | 1 | 2 | 1 | 1 | 1 | 9 |
| Stavrou EP 2010 | 1 | 1 | 1 | 1 | 2 | 1 | 0 | 1 | 8 |
| Suzuki H 2010 | 1 | 1 | 1 | 1 | 2 | 1 | 1 | 1 | 9 |
| Wouters MW 2009 | 1 | 1 | 1 | 1 | 1 | 1 | 1 | 1 | 8 |
| Kuo EY 2001 | 1 | 1 | 1 | 1 | 2 | 1 | 1 | 1 | 9 |
| Sheetz KH 2019 | 1 | 1 | 1 | 1 | 2 | 1 | 1 | 1 | 9 |
| Fischer C 2017 | 1 | 1 | 1 | 1 | 2 | 1 | 1 | 1 | 9 |
| Dimick JB 2005 | 1 | 1 | 1 | 1 | 2 | 1 | 1 | 1 | 9 |
| McCulloch P 2003 | 1 | 1 | 1 | 1 | 1 | 1 | 1 | 1 | 8 |
| Wenner G 2005 | 1 | 1 | 1 | 1 | 2 | 1 | 1 | 1 | 9 |
| Gillison EW 2002 | 1 | 1 | 1 | 1 | 2 | 1 | 1 | 1 | 9 |
| Ulrich G 2017 | 1 | 1 | 1 | 1 | 2 | 1 | 1 | 1 | 9 |
| Gasper WJ 2009 | 1 | 1 | 1 | 1 | 2 | 1 | 1 | 1 | 9 |
| Urbach DR 2005 | 1 | 1 | 1 | 1 | 2 | 1 | 1 | 1 | 9 |
| Rouvela L 2007 | 1 | 1 | 1 | 1 | 2 | 1 | 1 | 1 | 9 |
| Dimick JB 2003 | 1 | 1 | 1 | 1 | 2 | 1 | 1 | 1 | 9 |
| Dimick JB 2005 | 1 | 1 | 1 | 1 | 2 | 1 | 1 | 1 | 9 |
| Swisher SG 2000 | 1 | 1 | 1 | 1 | 1 | 1 | 1 | 1 | 8 |
| Munasinghe A 2015 | 1 | 1 | 1 | 1 | 2 | 1 | 1 | 1 | 9 |
| Nimptsch U 2017 | 1 | 1 | 1 | 1 | 2 | 1 | 1 | 1 | 9 |
| Steyerberg EW 2006 | 1 | 1 | 1 | 1 | 2 | 1 | 1 | 1 | 9 |
| Nimptsch U 2016 | 1 | 1 | 1 | 1 | 2 | 1 | 1 | 1 | 9 |
| Finley CJ 2011 | 1 | 1 | 1 | 1 | 2 | 1 | 1 | 1 | 9 |
| Leigh Y 2009 | 1 | 1 | 1 | 1 | 2 | 1 | 1 | 1 | 9 |
| Wouters MW 2008 | 1 | 1 | 1 | 1 | 2 | 1 | 1 | 1 | 9 |
| Ra J 2008 | 1 | 1 | 1 | 1 | 2 | 1 | 1 | 1 | 9 |
| Allaredd V 2007 | 1 | 1 | 1 | 1 | 2 | 1 | 1 | 1 | 9 |
| Simunovic M 2006 | 1 | 1 | 1 | 1 | 2 | 1 | 1 | 1 | 9 |
| Dimick JB 2003 | 1 | 1 | 1 | 1 | 1 | 1 | 1 | 1 | 8 |
| Urbach DR 2003 | 1 | 1 | 1 | 1 | 2 | 1 | 1 | 1 | 9 |
| Dikken JL2013 | 1 | 1 | 1 | 1 | 2 | 1 | 0 | 1 | 8 |
| Rodgers M 2007 | 1 | 1 | 1 | 1 | 2 | 1 | 0 | 1 | 8 |
| Markar S 2015 | 1 | 1 | 1 | 1 | 2 | 1 | 1 | 1 | 9 |
| Voeten DM 2021 | 1 | 1 | 1 | 1 | 2 | 1 | 1 | 1 | 9 |
| Kennedy GT 2018 | 1 | 1 | 1 | 1 | 2 | 1 | 1 | 1 | 9 |
| Sakata R 2012 | 1 | 1 | 1 | 1 | 2 | 1 | 1 | 1 | 9 |
| Allareddy V 2010 | 1 | 1 | 1 | 1 | 2 | 1 | 1 | 1 | 9 |

**Supplemental Data Figure 1.** Forest Plot of Association Between Hospital Surgical Case Volume and Risk of Postoperative Mortality Among Esophageal Cancer Patients Undergoing Esophagectomy According to Country, Eastern Versus Western.

**Supplemental Data Figure 2.** Forest Plot of Association Between Hospital Surgical Case Volume and Risk of Postoperative Mortality Among Esophageal Cancer Patients Undergoing Esophagectomy According to Study Period, 1984–2004 Versus 2005–2019.

**Supplemental Data Figure 3.** Forest Plot of Association Between Hospital Surgical Case Volume and Risk of Postoperative Mortality Among Esophageal Cancer Patients Undergoing Esophagectomy According to Hospital Number, ≤100, ≥100 and Unkown.

**Supplemental Data Figure 4.** Forest Plot of Association Between Hospital Surgical Case Volume and Risk of Postoperative Mortality Among Esophageal Cancer Patients Undergoing Esophagectomy According to Sample Size, ≤5000, ≥5000 and Unkown.

**Supplemental Data Figure 5.** Forest Plot of Association Between Hospital Surgical Case Volume and Risk of Postoperative Mortality Among Esophageal Cancer Patients Undergoing Esophagectomy According to Adjusted Factor, Yes Versus Unknown.

**Supplemental Data Figure 6.** Forest Plot of Association Between Hospital Surgical Case Volume and Risk of Postoperative Mortality Among Esophageal Cancer Patients Undergoing Esophagectomy According to Volume Grouping, Dichotomous, Tertiles, Quartiles, Quintiles, Sextiles, and Seventh Percentiles.

**Supplemental Data Figure 7.** Forest Plot of Association Between Hospital Surgical Case Volume and Risk of Postoperative Mortality Among Esophageal Cancer Patients Undergoing Esophagectomy According to NOS Quality Assessments, Total Score = 9 versus Total Score < 9.

**Supplemental Data Figure 8.** Forest Plot of Association Between Hospital Surgical Case Volume and Risk of Postoperative Mortality Among Esophageal Cancer Patients Undergoing Esophagectomy According to NOS Quality Assessments, Comparability Score = 2 versus Comparability Score < 2.

**Supplemental Data Figure 9.** Forest Plot of Leave-One-Out Sensitivity Analysis.
